# *Pyemotes ventricosus* Dermatitis, Southeastern France 

**DOI:** 10.3201/eid1411.080288

**Published:** 2008-11

**Authors:** Pascal Del Giudice, Véronique Blanc-Amrane, Philippe Bahadoran, Eric Caumes, Pierre Marty, Mariléna Lazar, Christian Boissy, François Desruelles, Arezki Izri, Jean-Paul Ortonne, Evelyne Counillon, Olivier Chosidow, Pascal Delaunay

**Affiliations:** Hôpital Bonnet, Fréjus, France (P. Del Giudice, M. Lazar, C. Boissy, E. Counillon); Centre Hospitalier d’Antibes-Juan les Pins, Antibes-Juan les Pins, France (V. Blanc-Amrane); Centre Hospitalier Universitaire de l’Archet, Nice, France (P. Bahadoran, P. Marty, F. Desruelles, J.-P. Ortonne, P. Delaunay); Hôpital Pitié–Salpêtrière, Paris, France (E. Caumes); Hôpital Avicenne, Paris, France (A. Izri); and Hôpital Tenon, Paris, France (O. Chosidow)

**Keywords:** Pyemotes ventricosus, mite, southern France, dispatch

## Abstract

We investigated 42 patients who had unusual pruritic dermatitis associated with a specific clinical sign (comet sign) in 23 houses in southeastern France from May through September 2007. *Pyemotes ventricosus,* a parasite of the furniture beetle *Anobium punctatum,* was the cause of this condition.

In 2006, we described an outbreak of unusual dermatitis in southeastern France (*1*). Patients affected had highly erythematous pruritic macules typical of arthropod bites, sometimes associated with a linear erythematous macular tract that we called the comet sign (Figure 1). The cause of this outbreak remained unknown. In May 2007, during an entomologic ecoenvironmental investigation conducted inside the homes of some of these patients, we found wooden furniture, which harbored live furniture beetles (*Anobium punctatum*) (Figure 2, panel A), and small amounts of wood dust on the floor. Because *A*. *punctatum* does not bite humans or cause contact dermatitis (*2*), it was not considered as the direct causative agent. However, stereomicroscope examination of the wood dust identified the mite *Pyemotes ventricosus* (Figure 2, panel B). Because *Pyemotes* spp. can cause dermatitis (*3*), they were considered as the hypothetical agent causing the eruption. We then conducted an observational and entomologic study of the new cases.

**Figure 1:**
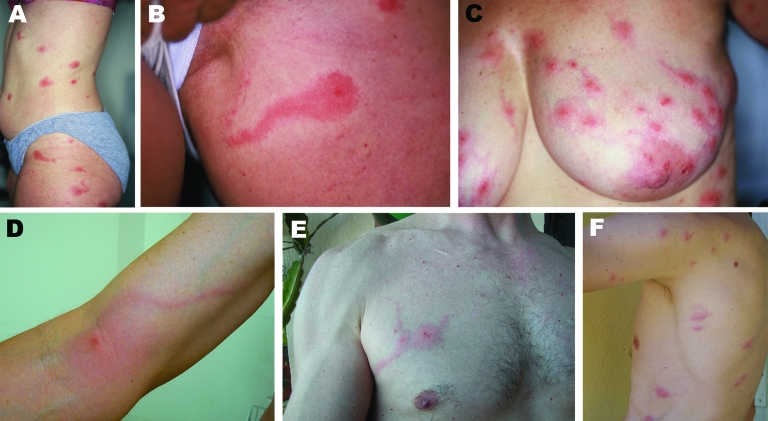
A–F) Photographs of 6 persons with skin lesions of *Pyemotes ventricosus* dermatitis. Note the central microvesicles, ulcerations or crusts, and some lesions with the comet sign. D) Lymphangitis-like dermatitis. E, F) Lesions resulting from natural infection of 2 of the investigators.

**Figure 2:**
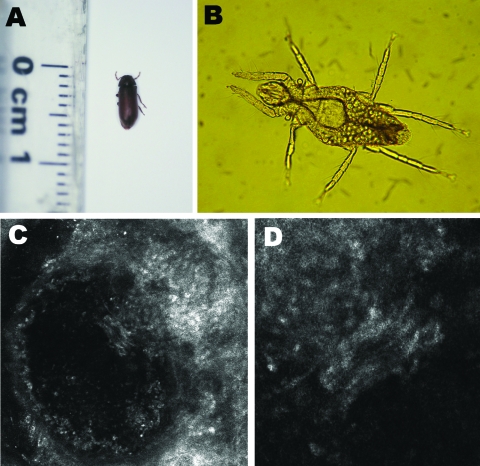
Organisms involved in transmission of *Pyemotes ventricosus* dermatitis. A) Common furniture beetle (*Anobium punctatum*) (5 × 2 mm). B) Nongravid female *P. ventricosus* mite (210 × 40 μm). C) Confocal laser scanning microscopy (CLSM) image (CLSM Vivascope 1500 microscope; Lucid Inc., Rochester, NY, USA) of a *P. ventricosus* mite (210 × 40 μm) in its microvesicle. D) Higher magnification of the microvesicle in panel C (light area in the center) (magnification ×4).

## The Study

From May through September 2007, all patients referred to the dermatology department at Fréjus Hospital (Fréjus, France) with suspected arthropod-bite dermatitis were examined; patients were also checked for the comet sign. Patients were asked about their outdoor activities (walking in the countryside, gardening), homes, and whether they had wooden furniture potentially infested with furniture beetles. Skin eruptions were photographed. Detailed clinical and histologic descriptions of the lesions have been reported (*1*).

Whenever possible, ecoenvironments (surrounding terrain and home interior) of patients with typical macules associated with the comet sign were investigated. Household pets were examined by veterinarians. When wormholes were found in wooden furniture, *A*. *punctatum* was noted as present in the immediate environment. Mites were collected and examined under a stereomicroscope (magnification ×80). Removal of furniture was recommended if it was infested with *A*. *punctatum*.

From May through October 2007, 42 patients with typical eruption formed 23 clusters, comprising 1–5 patients each living in the same home. Nineteen patients lived <50 km from Fréjus and 23 were vacationing in this city. Among the 23 homes in the cluster, 14 were entomologically and ecoenvironmentally investigated. *A*. *punctatum* (Figure 2, panel A) was found in all 14 homes and *P*. *ventricosus* was found in 12 (83%). Females (Figure 2, panel B), some gravid, and rare males were seen. Entomologic investigations outside homes found no evidence of any other insect pests. Veterinarians who examined the 2 pets living in these homes excluded dog ectoparasites. Dermatitis persisted or recurred for weeks in all patients until infested furniture was removed or patients left their homes. Oral prednisone (0.5 mg/kg) rapidly (within 48 hours) attenuated the pruritus.

One of us (P.D.) volunteered to place *P*. *ventricosus*–infested wood dust on 1 cm^2^ of abdominal skin under an occlusive bandage for 4 hours. Two negative controls consisted of placing uncontaminated wood dust under a bandage and the bandage alone. Twenty-four hours later, an erythematous macular pruritic lesion was observed that lasted for 7 days, but no comet sign developed. No cutaneous reaction was observed under the 2 negative-control bandages.

Natural dermatitis also occurred in 2 investigators during collection of ecoenvironmental samples. Typical lesions with the comet sign (Figure 1, panels E and F) developed in these investigators after they touched wood dust and infested furniture (1 of the investigators had only entered a room with infested furniture but had not touched it). Skin scrapings of the investigators did not contain parasites. A skin-biopsy specimen from 1 investigator showed a subcorneal ulceration but no parasite. Skin lesions were examined dermoscopically (magnification ×40) and showed a microulceration or vesicle in the center of the macule. In contrast, in vivo confocal laser scanning microscopy (CLSM) of a microvesicle from 1 investigator showed an ovoid foreign body with morphologic features suggestive of *P*. *ventricosus* (Figure 2, panels C and D).

## Conclusions

In 2006, we described an outbreak of an unusual dermatitis associated with a specific clinical sign that we called the comet sign (*1*). In 2007, similar cases occurred and we demonstrated that the mite *P*. *ventricosus* was the causative agent of the condition. *P*. *ventricosus* (Newport, 1850) (phylum Arthropoda, class Arachnida, order Acarina, suborder Prostigmata, family Pyemotidae) is an ectoparasite of arthropod larvae. This mite has been reported to be associated with *A*. *punctatum* (De Geer, 1774), the common furniture beetle (*2*).

This mite was associated with 2 investigators’ macular lesions that were acquired during experimental or natural infection. Natural infestation gave rise to macules frequently associated with the comet sign. We visualized, by using in vivo CLSM, a microvesicle, an ovoid foreign body with morphologic features suggestive of the mite. An entomologic ecoenvironmental investigation found *P*. *ventricosus* and its host (*A*. *punctatum*) in 83% of the patients’ homes investigated. Although *Pyemotes* spp.–related dermatitis outbreaks have been described, the outbreak we describe showed an emergent pattern with documented intradomiciliary infestation and the comet sign.

Although *Pyemotes* spp. have been known since the beginning of the 20th century to cause dermatitis (*5*), recent reports are scarce. In all recorded outbreaks, ectoparasites of insect larvae feeding on plants were responsible for dermatitis in workers exposed to agricultural products (*3*–*7*). Recent reports of dermatitis caused by *Pyemotes* spp. are even rarer (14 clinical reports since 1961), which make this dermatitis an almost forgotten disease (*8*–*13*).

The outbreak was associated with home interior infestations of *P*. *ventricosus* associated with *A*. *punctatum.* Fine and Scott described the first cases of dermatitis caused by *P*. *ventricosus* parasitizing *A*. *punctatum* (*8*). In Great Britain, Hickin found that up to 60% of woodworm larvae in damp locations were parasitized with *Pyemotes* spp., which were considered responsible for some skin irritations in woodsmen (*2*). More recently, Rodriguez-Casado et al. (*11*) described a *P*. *dermatitis* outbreak associated with *A*. *punctatum*–infested wood desks in a school.

Our cases were concentrated from May through September. The fact that we observed most cases in July and August might reflect the mite’s life cycle, which is activated when temperatures reach 80°F (26°C) (*3*). Moreover, many patients (23/42 in 2007) were on summer vacation, and most of them were living in homes that had been closed for months (i.e., not cleaned regularly), making it likely that the interior mite concentration would be high.

*Pyemotes* spp. dermatitis has been described as a pruritic erythematous rash with maculopapules with a central microvesicle (*7*). We initially reported that the typical pruritic erythematous macules were sometimes associated with a linear tract (comet sign) (*1*). Several hours after exposure, a linear erythematous macular tract arose from patients’ lesions. Whether this sign is specific to *Pyemotes* spp. or *P*. *ventricosus* remains unknown. The epidermis along the linear tract was clinically and histologically intact (*1*), thereby making contact dermatitis or epidermal migration of *P.* *ventricosus*, as in human scabies, unlikely. In vivo CLSM detected features suggestive of the mite inside the cutaneous microvesicle. A comet sign might represent onset of specific lymphangitis, as suggested for 1 patient (Figure 1, panel D). Two recent similar cases in southern France, which were considered specific atypical lymphangitis (*14*,*15*), might have been *P*. *ventricosus* dermatitis.

## References

[1] Del Giudice P, Caumes E, Boissy C, Leduff F, Delaunay P, Blanc-Amrane V, An outbreak of creeping eruption in southern France. Br J Dermatol. 2007;157:824–5. 10.1111/j.1365-2133.2007.08085.x 17655738

[2] Hickin E. The woodworm. Science Journal. 1969;3:64–70.

[3] Hewitt M, Barrow GI, Miller DC, Turk SM. A case of *Pyemotes* dermatitis, with a note on the role of these mites in skin disease. Br J Dermatol. 1976;94:423–30. 10.1111/j.1365-2133.1976.tb06120.x 1268056

[4] Cross EA, Moser JC. A new dimorphic species of *Pyemotes* and key to previously described forms (Acarina: Tarsonemoidea). Ann Entomol Soc Am. 1975;68:723–32.

[5] Letchford J, Strungs I, Farrell D. *Pyemotes* species strongly implicated in an outbreak of dermatitis in a Queensland country hospital. Pathology. 1994;26:330–2. 10.1080/00313029400169771 7991293

[6] Swan DC. The hay itch mite *Pediculoides ventricosus* (Newport) (Acarina, Pediculoidae) in South Australia. Journal of Agricultural India and South Australia. 1934;37:1289–99.

[7] Booth BH, Jones RWBB. Epidemiological and clinical study of grain itch. JAMA. 1952;150:1575–9. 10.1001/jama.1952.03680160025006 12990473

[8] Fine RM, Scott HG. Straw itch mite dermatitis caused by *Pyemotes ventricosus*: comparative aspects. South Med J. 1965;58:416–20. 14276420 10.1097/00007611-196504000-00003

[9] Rycroft RJ, Kennedy C. *Pyemotes dermatitis* in display artists. Clin Exp Dermatol. 1981;6:629–34. 10.1111/j.1365-2230.1981.tb02368.x 6210471

[10] Betz TG, Davis BL, Fournier PV, Rawlings JA, Elliot LB, Baggett DA. Occupational dermatitis associated with straw itch mites (*Pyemotes ventricosus*). JAMA. 1982;247:2821–3. 10.1001/jama.247.20.2821 6210784

[11] Rodríguez-Casado MJ, Cerro-González R, Martín-Blázquez JL, Vázquez-Contioso M. Outbreak of *Pyemotes dermatitis* in an elementary school [in Spanish]. Enferm Infecc Microbiol Clin. 2004;22:370–1. 10.1157/13063055 15228911

[12] Broce AB, Zurek L, Kalish JA, Brown R, Keith DL, Gordon D, *Pyemotes herfsi* (Acari: Pyemotidae), a mite new to North America as the cause of bite outbreaks. J Med Entomol. 2006;43:610–3. 10.1603/0022-2585(2006)43[610:PHAPAM]2.0.CO;2 16739423

[13] Centers for Disease Control and Prevention. Outbreak of pruritic rashes associated with mites—Kansas, 2004. MMWR Morb Mortal Wkly Rep. 2005;54:952–5. 16195693

[14] Abraham S, Tschanz C, Krischer J, Saurat JH. Lymphangitis due to insect sting. Dermatology. 2007;215:260–1. 10.1159/000106587 17823527

[15] Marque M, Girard C, Guillot B, Bessis D. Dermite érythémateuse linéaire supra-lymphatique réactionnelle: une nouvelle entité clinico-histologique (poster no. 121). In: Abstracts of the Paris Dermatology Meeting. 5–9 December 2006, Paris, France. Ann Dermatol Venereol. 2006;133:4S1–298.

